# Cofactor Specificity Engineering of *Streptococcus mutans* NADH Oxidase 2 for NAD(P)^+^ Regeneration in Biocatalytic Oxidations

**DOI:** 10.5936/csbj.201402005

**Published:** 2014-02-26

**Authors:** Barbara Petschacher, Nicole Staunig, Monika Müller, Martin Schürmann, Daniel Mink, Stefaan De Wildeman, Karl Gruber, Anton Glieder

**Affiliations:** a Austrian Centre of Industrial Biotechnology GmbH, c/o Institute of Molecular Biotechnology, Graz University of Technology, Petersgasse 14, 8010 Graz, Austria; b Austrian Centre of Industrial Biotechnology GmbH, c/o Institute of Molecular Biosciences, University Graz, Humboldtstrasse 50/3, 8010 Graz, Austria; c DSM Innovative Synthesis B.V., P.O. Box 18, 6160 MD Geleen, Netherlands

**Keywords:** coenzyme selectivity, NADPH recycling, 2-heptanol oxidation, NADPH oxidase, site-directed mutagenesis, cofactor regeneration

## Abstract

Soluble water-forming NAD(P)H oxidases constitute a promising NAD(P)^+^ regeneration method as they only need oxygen as cosubstrate and produce water as sole byproduct. Moreover, the thermodynamic equilibrium of O_2_ reduction is a valuable driving force for mostly energetically unfavorable biocatalytic oxidations. Here, we present the generation of an NAD(P)H oxidase with high activity for both cofactors, NADH and NADPH. Starting from the strictly NADH specific water-forming *Streptococcus mutans* NADH oxidase 2 several rationally designed cofactor binding site mutants were created and kinetic values for NADH and NADPH conversion were determined. Double mutant 193R194H showed comparable high rates and low *K*_m_ values for NADPH (*k*_cat_ 20 s^-1^, *K*_m_ 6 µM) and NADH (*k*_cat_ 25 s^-1^, *K*_m_ 9 µM) with retention of 70% of wild type activity towards NADH. Moreover, by screening of a SeSaM library *S. mutans* NADH oxidase 2 variants showing predominantly NADPH activity were found, giving further insight into cofactor binding site architecture. Applicability for cofactor regeneration is shown for coupling with alcohol dehydrogenase from *Sphyngobium yanoikuyae* for 2-heptanone production.

## Introduction

Enzyme catalyzed oxidation reactions have gained increasing interest in biocatalysis recently, reflected also by a number of excellent reviews on this topic published in the last years [1–3]. Oxidoreductases constitute an important group of biocatalysts as they facilitate not only the widely used stereoselective reduction of aldehydes and ketones but also the less well exploited oxidation of alcohols and amines. Oxidoreductases catalyzed oxidations are also used for production of chiral alcohols and amines by deracemization [1, 4–6].

Oxidoreductases, especially aldo-keto-reductases and dehydrogenases, act on the substrate by the transfer of electrons from or to a cofactor, mostly the nicotinamide-based nucleotides NAD(H) and NADP(H). As nicotinamide cofactors are expensive, regeneration of cofactors is necessary for economically feasible biocatalytic processes. While for the regeneration of the reduced cofactors NADH and NADPH several systems (engineered formate dehydrogenase [7, 8], phosphite dehydrogenase [9, 10], glucose dehydrogenase [11, 12] plus cosubstrate) are well established and widely used, universal regeneration systems for the oxidized forms NAD^+^ and NADP^+^ are less well developed.

Enzyme based, electrochemical, chemical, and photochemical regeneration methods are known. Coupled substrate or coupled enzyme systems [4, 13, 14] constitute two possibilities for enzymatic NAD(P)^+^ recycling. In these reaction set-ups the cofactor is regenerated via the reduction of a carbonyl group of a cosubstrate, catalyzed either by the production enzyme itself (coupled substrate) [15] or by an additionally added dehydrogenase (coupled enzyme; glutamate dehydrogenase [16, 17], lactate dehydrogenase [18]). Carbonyl cosubstrate reductions by dehydrogenases normally provide little thermodynamic driving force for mostly energetically unfavorable biocatalytic alcohol oxidations. Generally, it is therefore necessary to supply the cosubstrate in excess to achieve high substrate conversion rates. In recent studies several smart concepts have been introduced to reduce the need for cosubstrate. The use of one-way cosubstrates [19] or cofactor regeneration as an integral part of a redox neutral multi-enzyme network [20, 21] was reported.

Several cofactor regeneration systems benefit from the high driving force of molecular oxygen as hydrogen acceptor. One example therefore is a 9,10 phenantrenequinone/xylose reductase system where the quinone is auto-reoxidized by oxygen [22]. O_2_ reduction also drives cofactor regeneration via mediators as ABTS or Meldola's blue, which are reoxidized by a laccase under H_2_O formation [23–25]. Instead of using laccase the mediator reoxidation can also be achieved by electrochemical means, albeit at moderate turnover numbers. To overcome the still rather low productivity of electrochemical regeneration processes careful reaction and cell design is necessary [26–28]. In pure chemical regeneration processes the chemical agent directly reoxidizes the cofactor without biocatalyst. Often Ruthenium complexes are used as oxidants [14]. The direct regeneration of NAD(P)^+^ via FMN was found to be strongly accelerated by light-induced excitation of FMN [29].

A very promising NAD(P)^+^ regeneration method is the application of soluble NAD(P)H oxidases (EC 1.6.3.1 NOX) from bacteria or archaea which use molecular oxygen as oxidant. This regeneration method has the advantage of being cheap as no cosubstrate or mediator is needed. Straightforward downstream processing is possible as only hydrogen peroxide or water is formed as byproduct. Moreover, the high redox potential of oxygen results in a high thermodynamic driving force. The electron and hydrogen transfer from NADH to oxygen is catalyzed by known soluble NAD(P)H oxidases via a two electron transfer producing hydrogen peroxide (equation 1) or a four electron transfer producing water (equation 2) [30, 31].

1 $$\text{NAD}(\text{P})\text{H}+{\text{O}}_{2}+{\text{H}}^{+}\to \text{NAD}{\left(\text{P}\right)}^{+}+{\text{H}}_{2}{\text{O}}_{2}$$

2 $$2\text{NAD}(\text{P})\text{H}+{\text{O}}_{2}+2{\text{H}}^{+}\to 2\text{NAD}{\left(\text{P}\right)}^{+}+2{\text{H}}_{2}\text{O}$$

The four electron transferring oxidases are the preferred choice for cofactor regeneration as they only form water as byproduct. In case of H_2_O_2_ production, catalase has to be added to the system to prevent enzyme damage by the peroxide. Water-forming NADH oxidases have been studied from several bacteria as *Streptococcus* [32–35], *Lactobacillus* [36–38], *Lactococcus* [39], *Clostridium* [40], *Serpulina* [41], *Leuconostoc* [42] and *Bacillus* [43]. NOX from *P. furiosus* [44] and *T. kodakarensis* [45] produce H_2_O and a significant level of H_2_O_2_ (72% for *Pf*NOX, 25% for *Tk*NOX). NAD(P)H oxidases belong to the pyridine nucleotide disulfide oxidoreductases (PNDOR) together with, among others, glutathione reductase and CoA-disulfide reductase [46]. NOX enzymes contain a single conserved redox-active cysteine that circulates between the thiol/thiolate and the sulfenic acid state during catalysis. Overoxidation of the cysteine leads to enzyme deactivation. Several NADH oxidases need FADH or DTT addition for optimal performance [36, 45, 47]. Enzymes with high specific activities (> 150 U/mg) were recently reported from *L. sanfranciscensis, L. plantarum*, *L. rhamnosus* and *S. pyogenes* [34, 37, 38, 48]. A drawback in using NADH oxidases for cofactor regeneration is that almost all water-forming NADH oxidases are specific for NADH. In wild type form only two water forming NOXs and one hydrogen peroxide forming NOX show activity with NADPH (around half / one third of activity with NADH depending on the NOX [38, 49, 50]. NOX 1299 from *T. kodakarensis* is the only NOX showing higher wild type activity with NADPH than with NADH but disadvantageously it produces high amounts of H_2_O_2_ [45]. NOX of *L. plantarum* was recently successfully mutated to accept NADPH but resulting in a simultaneous decrease in NADH activity [37]. The *k*_cat_ value for NADH reaction of the variant with highest catalytic efficiency with NADPH was six-fold reduced compared to the wild type.

In this study we aimed at developing an NAD(P)H oxidase which is universally applicable for regeneration of NADH as well as NADPH. As starting point NADH specific water-forming *S. mutans* NADH oxidase 2 (*Sm*NOX) was chosen. *Sm*NOX was chosen as it was well characterized to be stable, highly active, not dependent on FADH or DTT addition [51], and had already been expressed in *E. coli* before [32]. Moreover, a crystal structure of a closely related enzyme from *S. pyogenes* was available which enabled us to model the *Sm*NOX structure. In a thorough mutation study of the cofactor binding site a *Sm*NOX mutant with matched activities with NADH and NADPH was generated. Mutants with increased NADPH/NADH activity ratios were identified by *Sm*NOX library screening. The conversion of 2-heptanol to 2-heptanone with NADPH regeneration by engineered *Sm*NOX was shown.

## Methods

### Strains and materials

*E. coli* TOP10F’ was originally bought from Invitrogen (Carlsbad, CA, USA), *E. coli* BL21-Gold (DE3) was from Stratagene (La Jolla, CA, USA). NAD(P)H was from Roche Diagnostics GmbH (Mannheim, Germany) or Roth (Karlsruhe, Germany). Materials for cloning were from Fermentas (St. Leon-Roth, Germany, now Thermo-Fisher Scientific) if not stated otherwise. All other chemicals were purchased from Sigma-Aldrich, Fluka (St. Luis, MO, USA) or Roth (Karlsruhe, Germany) if not stated otherwise.

### Homology modeling of S. mutans NADH oxidase 2

Homology modeling for *Streptococcus mutans* NOX 2 was based on an X-ray structure of NADH oxidase from *Streptococcus pyogenes* (template: 2BC0A, 2.00 Å). The sequence identity between target and template was 77.5%. The homology model was created with the automated protein structure homology-modeling server SWISS-MODEL developed by the Protein Structure Bioinformatics group at the SIB - Swiss Institute of Bioinformatics and the Biozentrum University of Basel (version February 2008) [52].

### Cloning and site directed mutagenesis

A synthetic *S. mutans* NOX 2 gene (protein sequence: GI:290580450) was ordered at DNA2.0 (Menlo Park, CA, USA) and ligated into a *Nde*I/*Hind*III cut pMS470Δ8 vector [53] downstream of the tac-promoter to give the vector pMSsN1Wt. Site directed mutagenesis of the *S. mutans* NOX 2 gene in vector pMSsN1Wt was performed following the Stratagene Quikchange Site-directed Mutagenesis Kit instruction (*Stratagene*, *La Jolla*, CA, USA). Primers used for site directed mutagenesis are listed in Table 1. PCR reaction mixtures (50 µL) contained template plasmid (28 pM), primers (0.2 µM each), dNTPs (200 µM each) and 10 x reaction buffer (5 µL) supplied with the polymerase. *Pfu* turbo polymerase (Stratagene, 2.5 units) was added to each tube. The amplification protocol comprised 30 seconds of initial denaturation at 95 °C, 18 cycles of denaturation (30 s, 95 °C), annealing (1 min, 55 °C) and extension (6 min, 68 °C), and a final 7 minutes extension period at 68 °C. After 1 h of *Dpn*I digestion competent *E. coli* TOP10F’ cells were electrotransformed with the reaction mixture (2 µL). Successful incorporation of the desired mutations was verified by dideoxy sequencing.

**Table 1:** Primers used in PCR for site directed mutagenesis of *S. mutans* NOX 2.

Mutated amino acidsa)	Forward primerb) (mismatched bases are underlined)
Asp192→Ala	5’-agaagttatcctgatcgccgttgttgacacctgcc-3’
Asp192→Arg	5’-aaagaagttatcctgatcaacgttgttgacacctgcc-3’
Val193→Arg	5’-gaagttatcctgatcgaccgtgttgacacctgcctggc-3’
Val194→His	5’-gttatcctgatcgacgttcatgacacctgcctggca-3’
Ala199→Arg	5’-gttgttgacacctgcctgcgtggttactacgaccaggac-3’
Gly200→Lys	5’-gttgacacctgcctggcaaaatactacgaccaggacctg-3’
Asp192→Ala/Val193→Arg	5’-taaagaagttatcctgatcgcccgtgttgacacctgcctggcag-3’
Val194→His/Ala199→Argc)	5’-gttcatgacacctgcctgcgtggttactacgaccaggac-3’
Val194→His/Gly200→Lysc)	5’-catgacacctgcctggcaaaatactacgaccaggacctg-3’

a) Numbering refers to *S. mutans* NOX sequence beginning with 1 for the starting methionine

b) reverse primers have reverse complementary sequence

c) for combination of mutations the plasmid carrying the Val194→His mutation was used as template

### Preparation of cell free extracts

Electrocompetent *E. coli* BL21 (DE3) Gold cells were transformed with plasmid pMSsN1Wt or one of 15 variants thereof. Additionally, a plasmid pMSsN2 was transformed into an *E. coli* BL21 (DE3) giving a strain overexpressing *Lactobacillus sanfranciscensis* NOX (*Ls*NOX). pMSsN2 is identical to pMSsN1 except that it carries the gene coding for *Ls*NOX (synthetic variant, ordered at DNA2.0, protein sequence: GI:11862874) instead of the *Sm*NOX. Precultures of all resulting *E. coli* strains were cultivated in LB media (50 mL) containing Ampicillin (100 mg/L) in baffled shake flasks (300 mL) at 37 °C and 130 rpm overnight. Main cultures were inoculated to an OD of 0.05 in the described medium (250 mL in 1 L baffled flasks) and cultivated at 37 °C and 130 rpm. NOX production was induced at OD 0.8-1 by addition of IPTG (1 mM). Cells were harvested after an overnight induction period (25 °C, 110 rpm) by centrifugation (15 minutes, 5000 rcf, Avanti J-20 XP centrifuge, Beckman Coulter, Krefeld, Germany, rotor JA-10). Cell pellets were diluted in potassium phosphate buffer (50 mM, pH 7.0) to a final volume of 25 mL. Cell breakage was achieved by ultrasonication with a Branson sonifier 250 (Branson ultrasonic corporation, Danbury, CT, USA) for 6 minutes at 50 W with continuous cooling, pulsed with one 700 ms pulse per second with a 1 cm diameter tip. Cell free lysates were prepared by collecting the supernatant of centrifugation at 36000 rcf (rotor JA-25.50) for 45 minutes and concentrating it to half the volume via Vivaspin 20 centrifugal concentration tubes with 30 kDa molecular weight cutoff (Sartorius, Göttingen, Germany). Cell free extracts from an *E. coli* strain expressing *S. yanoikuyae* ADH (*Sy*ADH) from the pEamTA based plasmid pEam_SyADH was prepared following the same protocol.

### Protein content determination, SDS-PAGE

The protein content was determined with bichinonic acid protein assay (BCA) kit (Thermo Scientific, Waltham, MA, USA) using BSA as standard. For SDS-PAGE NuPAGE^®^ 4-12% Bis-Tris Gels, 1.0 mm, from Invitrogen, (Carlsbad, CA, USA) were used with a NuPAGE MOPS SDS Running Buffer for Bis-Tris Gels. All strains were stored as glycerol stocks at -80 °C. Cell free extracts were stored in aliquots at -20 °C.

### Enzyme activity assay

Initial rate data of NAD(P)H oxidation were acquired measuring the decrease in NAD(P)H absorption at 340 nm (ε= 6220 M^-1^ cm^-1^) in potassium phosphate buffer (50 mM, pH 7.0) at 25 °C. The tempered buffer was vortexed for saturation with oxygen before mixing with the enzyme and cofactor solution. Absorption measurements were performed on a Spectramax Plus 384 (Molecular Devices, Sunnyvale, CA, USA) or on a Synergy MX (Biotek, Winooski, VT, USA) in UV-star micro titer plates (Greiner, Kremsmünster, Austria). The total reaction volume was 200 µL, reactions were started by addition of NAD(P)H. Data collection was started after 5 seconds of mixing. For activity measurements with FAD, enzyme preparations first were pre-incubated in 10 fold concentration in 50 mM KP_i_ containing 25 µM or 250 µM FAD and then were diluted 1:10 in the same buffer for initial rate detection after addition of NADH. In case of DTT addition the assay buffer contained 5 mM of DTT.

### Determination of catalytic constants for SmNOX variants

Apparent kinetic parameters were obtained from initial rate measurements at air saturation oxygen level (250 µM) with eight cofactor concentrations varying over a concentration range of 5 to 10 times the apparent *K*_m_ or to a maximum NAD(P)H concentration of 1 mM. Enzymes were applied as crude lysates in dilutions chosen to give rates between 0.001 and 0.05 ▵Abs/min and rates were constant for ≥ 1 minute. Appropriate controls containing crude lysate without overexpressed NOX verified that blank rates were insignificant for all conditions used. Results from initial rate measurements were fitted to a Michaelis-Menten type equation (equation 3) using unweighted least-squares regression analysis performed with Sigmaplot program version 11 (Systat Software Inc.).

3 $$\text{v}={\text{v}}_{\max}*\text{A}/({\text{K}}_{\text{m}}+\text{A})$$

*v* is the initial rate, *v*_max_ is the apparent maximum rate (U/mg total protein in cell free extract), A the cofactor concentration and *K*_m_ the apparent Michaelis constant for NAD(P)H at air saturation oxygen levels.

### H_2_O_2_ determination

Enzyme assay reaction mixtures with 1 mM of NAD(P)H were fully converted with purified *Sm*NOX wild type or variant 193R194H. The assay solution or glucose standard solutions were diluted 1 + 1 with an *o*-dianisidine / glucose oxidase / horse radish peroxidase mixture [54] and absorption was measured at 460 nm.

### Recloning in pEHISTEV vector and enzyme purification

*Sm*NOX wild type gene and variant 193R194H were recloned in pEHISTEV vector [55] via *Eco*RV/*Hind*III restriction sites. A pEHISTEV version was used in which a second *Eco*RV restriction site was eliminated by introduction of a silent mutation. Plasmid preparations checked for correct sequence were transformed into *E. coli* BL21 (DE3) Star. Expression and cell free extract preparation were done as described above but in LB/Kanamycin medium. The cells were harvested by centrifugation (5000 g, 15 minutes). The pellet was resuspended in 50 mM KP_i_ pH 7.0 and disrupted by ultrasonication. The cell free extract was applied to a 5 mL Ni-Sepharose 6 Fast Flow column (GE Healthcare, Chalfont St Giles, UK). The tagged enzymes were obtained by a one-step purification using the buffers recommended in the manual. After purification, the enzyme buffer was exchanged to 50 mM KP_i_ and enzymes were concentrated to protein concentrations above 5 mg/mL by Vivaspin 20 tubes with 10 kDa molecular weight cutoff (Sartorius) before storage at -20 °C. The 6xHis tag was cleaved off from 1 mg NOX by incubation with tobacco etch virus protease (TEV protease) in a reaction mixture containing 0.2 mM EDTA, and 1 mM DTT in 50 mM Tris/HCl buffer, pH 8.0 by overnight incubation at 4 °C. 10 µg TEV protease were used per mg NOX. The mixture was applied on the Ni-Sepharose 6 Fast Flow column and washed through with 15 mL of 30 mM sodium phosphate buffer containing 0.3 M NaCl and 20 mM imidazole, pH 7.5. The flow through was collected. Buffer exchange to 50 mM KP_i_ samples and concentration to > 1 mg/mL protein was done in Vivaspin 20 centrifugal concentrators (Satorius AG, Göttingen, Germany), aliquots of concentrated NOX solutions were stored at -20 °C.

### Library generation and cultivation

A sequence saturation mutagenesis library [56] of *Sm*NOX gene with random mutations was bought at SeSaM Biotech (Aachen, Germany). The library was based on mutant *Sm*NOX 194H200K. The library was cloned into pMS470 vector and transformed into *E. coli* TOP10F’. Transformants were picked into 60 µL LB/Ampicillin media in 384 well plates, grown overnight at 37 °C and 60% of humidity and stored as 15% glycerol stocks at -80 °C. Cultivation of the expression library was done in 96 well plate format. Preculture plates with 150 µL of LB/Ampicillin media per well were inoculated from glycerol stock plates and cultivated at 37 °C at 60% humidity for at least 12 hours. Main culture plates with V-shaped bottom contained 80 µL of LB/Ampicillin media and were inoculated from the preculture plates. After 8 hours of growth at 37 °C and 60% humidity *Sm*NOX2 expression was induced by addition of 20 µL of a 0.5 mM IPTG solution in LB/Ampicillin media. The plates were kept at 28 °C and 60% humidity for 16 hours. Cells were harvested by 15 minutes of centrifugation at 2500 g. Supernatant was decanted and the cell pellets were frozen at -20 °C for at least two hours.

### Screening of SmNOX variants from a random mutagenesis library for enhanced NADPH/NADH activity

Screening assays were carried out in 96 well plates. After thawing cell lysis was accomplished by addition of 100 µL lysis buffer (50 mM KP_i_, pH 7.0, 1 mg/mL lysozyme) and an 1 h incubation at 28 °C at 600 rpm. Cell debris was separated by centrifugation at 2500 g for 15 min at 4 °C. The supernatant was diluted 1 + 1 with 50 mM KP_i_, pH 7.0 and used for screening assays. 140 µL of 50 mM KP_i_, pH 7.0, were added to 10 µL of diluted supernatant in two plates in parallel. Reactions were started by addition of 50 µL of a 0.8 mM NADH or NADPH solution. Initial rates of NADH and NADPH conversion were measured by detection of decrease in absorption at 340 nm over three minutes. Activity with NADH and NADPH was compared for each well. 2800 clones were screened, 480 thereof were chosen for a re-screen and the best 40 thereof were measured in a re-re-screen. Best variants were finally cultivated in shake flasks. From best variants plasmid DNA was isolated with Gene JetTM Plasmid Miniprep Kit (Fermentas, St. Leon-Roth, Germany) and sent for sequencing.

### Conversion experiments

Conversion experiments were set up in 1.5 mL reaction tubes. The reaction mixture contained NADP^+^ (100 µM) and 2-heptanol (10 mM) in potassium phosphate buffer (50 mM, pH 7.0) in a total volume of 500 µL. *Sphingobium yanoikuyae* ADH (*Sy*ADH) was applied as crude *E. coli* lysate and *Sm*NOX 193R194H was applied as purified enzyme in amounts to give 1 U/mL. After 12 hours at 25 °C and 600 rpm 100 µL of n-butanol (50 mM) was added as internal standard for GC analysis and the mixture was extracted with ethyl acetate (500 µL). Substrate conversion was determined by GC-analysis on a Varian CP7503 gas chromatograph equipped with an FID detector (300 °C) and a Phenomenex ZB-FFAP column (30 m x 0.32 mm; 0.25 µM) with a Restek Hydroguard MT precolumn (5 m x 0.32 mm). H_2_ was used as carrier gas (2.7 mL/min). The following temperature program was used: 65 °C to 110 °C, 9 °C/min; 110 °C to 160 °C, 25 °C/min. The retention time for 2-heptanol was 2.48 min and for 2-heptanone 3.44 min.

## Results and Discussion

### Mutation Strategy

**Figure 1:**
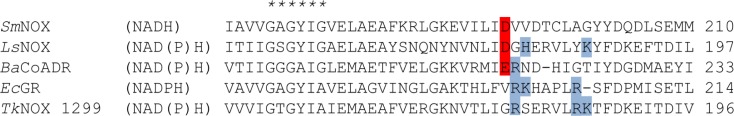
**Sequence alignment for the NAD(P)H-binding motifs of selected pyridine nucleotide disulfide oxidoreductases**. *Sm*NOX: *S. mutans* NADH oxidase 2, *Ls*NOX: *L. sanfranciscensis* NAD(P)H oxidase, *Ba*CoADR: *B. anthracis* CoA-disulfide reductase, *Ec*GR: *E. coli* Glutathione reductase, *Tk*NOX 1299: *T. kodakarensis* NAD(P)H oxidase, cofactor specificities given in brackets, red: negatively charged residues typical for NADH converting enzymes, blue: positively charged residues typical for NADPH converting enzymes, conserved sequence motif GXGXXG/A for dinucleotide binding marked with *, alignment was done with ClustalW2.

Water-forming *Streptococcus mutans* NADH oxidase 2 (*Sm*NOX) is a monomeric 50 kDa enzyme which is NADH specific [51]. We intended to establish *Sm*NOX as universal NAD(P)^+^ regeneration system by engineering the NADH specific wild type towards the effective usage of both cofactors, NADH and NADPH. Ideally, the created variant should have comparable characteristics for both cofactors to simplify application for cofactor regeneration in industrial processes with varying oxidizing enzymes. In NOX enzymes the nicotinamide cofactor is bound in the well described Rossmann fold manner [57]. In Rossmann fold enzymes often an acidic residue, typically an aspartate at the C-terminus of the second β-strand of the alternating βαβαβ-regions plays a key role in NAD(H) binding [58] by forming hydrogen bonds to the 2’-OH and 3’-OH of the adenine ribose. In contrast, NADP(H) specific Rossmann fold enzymes typically miss this acidic residue and instead carry a basic residue at the following amino acid position. Positive charges in the cofactor binding site facilitate the binding of the negatively charged phosphate group present in NADP(H) but not in NAD(H). The relevance of the described positions for cofactor specificity has first been shown in a mutation study of glutathione reductase by Scrutton et al. [58]. NADH oxidase from *Lactobacillus sanfranciscensis* (*Ls*NOX) exhibits a limited NADPH activity in parallel to NADH activity [38]. An alignment of the *Sm*NOX sequence with the glutathione reductase, *B. anthracis* coenzyme A-disulfide reductase and *Ls*NOX sequence (Figure 1) indicates several positions of possible importance for NADPH activity. *Sm*NOX and *Ls*NOX show an aspartate at the expected position indicative for NADH activity (position 192 in *Sm*NOX numbering including the starting methionine). The positive charge important for NADPH activity is missing at the following amino acid in both enzymes but in *Ls*NOX at the +2 position counted from the aspartate a positively charged histidine is found. Position 196 and 200 are also occupied by positively charged residues. Like *Ls*NOX, *B. anthracis* coenzyme A-disulfide reductase with dual cofactor specificity shows the negatively charged residue (here glutamate) in combination with a positively charged residue. The subtle side chain rearrangements and reformation of hydrogen bonds enabling the dual cofactor specificity of *B. anthracis* coenzyme A-disulfide reductase were recently described [59].

**Figure 2:**
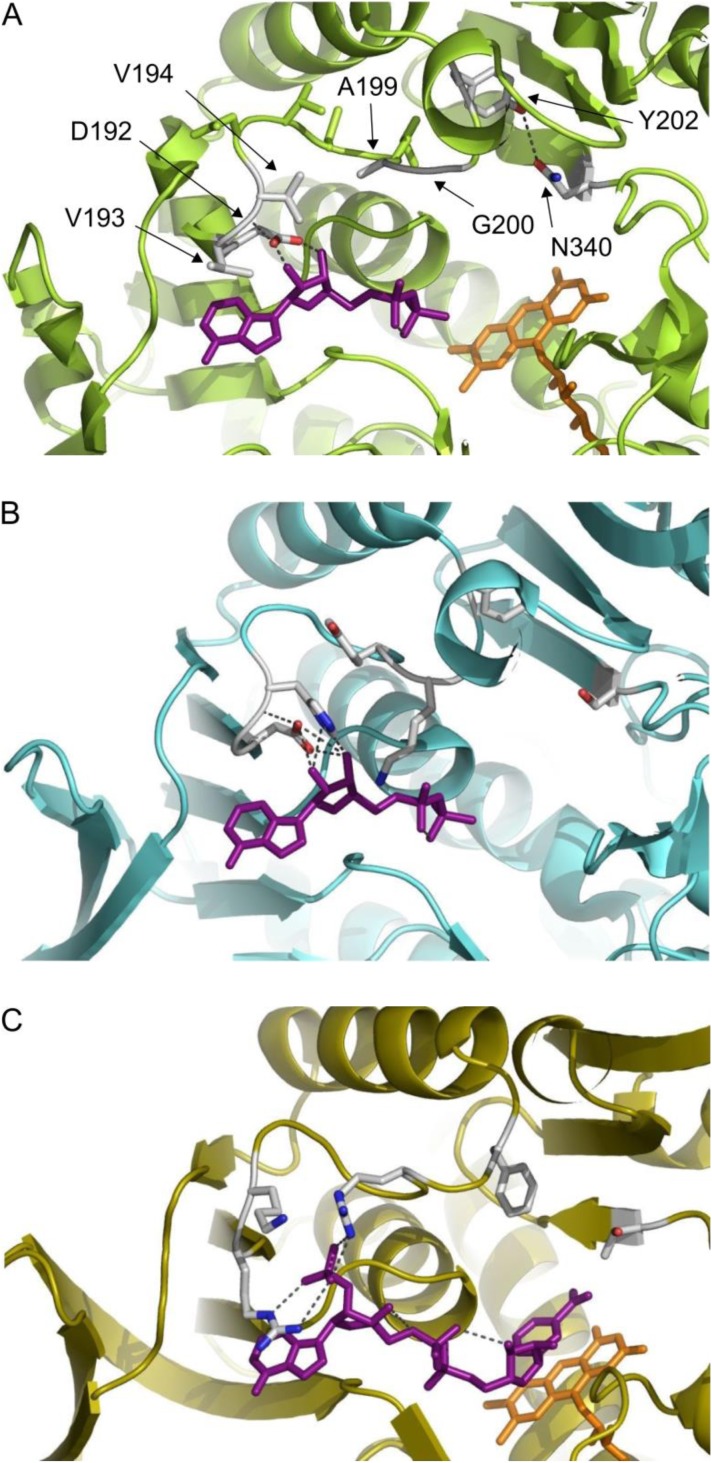
**Cofactor binding sites of *S. mutans* NADH oxidase 2, *L. sanfranciscensis* NAD(P)H oxidase and *E. coli* Glutathione reductase**. Panel A: Swiss-model for *S. mutans* NOX 2, based on *S. pyogenes* NOX structure (77.5% identity, pdb 2BC0), ADP from aligned *Ls*NOX structure pdb 2CDU, FAD from pdb 2BC0; panel B: *L*. *sanfranciscensis* NAD(P)H crystal structure pdb 2CDU with ADP bound [60]; panel C: *E. coli* Glutathione reductase crystal structure pdb 1GET [61], images were generated with PyMOL (Schrodinger Inc.)

For *Sm*NOX we built a structural model based on the X-ray structure of NADH oxidase from *S. pyogenes* (77.5% identity, 2BC0A). A comparison of the cofactor binding site of *Sm*NOX, *Ls*NOX, and *E. coli* glutathione reductase (Figure 2) indicated that amino acid residues at *Sm*NOX position 192-194 and 199-200 might possibly interact with the cofactor, while residues at position 195-198 are turned away from the cofactor. Positions 192-194, 199 and 200 were chosen for rational engineering of *Sm*NOX with the main aim of introducing positively charged residues to enable NADPH activity. In addition, single mutations were combined to double, triple, and quadruple mutants.

### Mutation and expression

**Figure 3:**
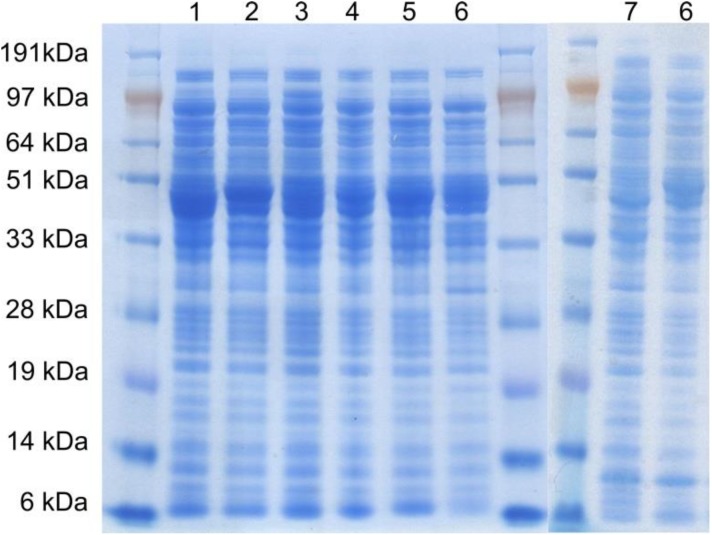
4-12% Bis-Tris SDS-PAGE gel of cell free extracts of *E. coli* BL21 (DE3) Gold expressing *Sm*NOX.

For recombinant expression and engineering, a synthetic *S. mutans* NOX 2 gene was inserted into pMS470Δ8 vector to give the vector pMSsN1Wt. Site directed mutagenesis of the *S. mutans* NOX 2 gene was performed following the Stratagene Quikchange protocol. Wild type and mutants were expressed in *E. coli* BL 21(DE3) Gold cells. Moreover, *Ls*NOX was expressed under the same conditions from vector pMSsN2, which is identical to pMSsN1Wt, except that it carries a synthetic gene for *Ls*NOX instead of the *Sm*NOX gene. SDS-PAGE gel analysis of cell free extracts indicated NOX over-expression for all variants with a strong band migrating to the expected position for 50 kDa. The band was not detected in the cell free extract of an *E. coli* BL21 strain without plasmid. In Figure 3 the SDS-PAGE analysis of cell free extracts for *Sm*NOX wild type and single mutants are shown.

### Apparent kinetic constants of cofactor specificity mutants

For *Sm*NOX and *Ls*NOX wild type and fourteen *Sm*NOX variants initial rate data for the oxidation of NADH or NADPH in cell free extracts at air saturated oxygen levels were recorded. Maximal specific activities and apparent *K*_m_ values were calculated by fitting velocities measured over a cofactor concentration range of up to 5-10 fold the *K*_m_ value to equation 3. Results are listed in Table 2. All mutants were cultivated under identical conditions and SDS-PAGE analysis showed a comparable expression level for all variants (see Figure 3).

**Table 2:** Apparent kinetic values and efficiencies for *Sm*NOX mutants and *Ls*NOX from cell free extracts.

		NADH			NADPH		
		
		*v*_max_ a) (U/mg)	*K*_m_ a) (µM)	*v*_max_/*K*_m_ (U/mg mMol)	*v*_max_ (U/mg)	*K*_m_ (µM)	*v*_max_/*K*_m_ (U/mg mMol)
	SmNOX Wt	3.5 ± 0.1	6 ± 1	580			0.09c)
	LsNOX	2.2 ± 0.1	9 ± 1	240	1.1 ± 0.1	5 ± 1	220
Mut1	D192Ab)	2.5 ± 0.1	18 ± 2	140	2.2 ± 0.1	140 ± 20	15
Mut2	D192N	2.8 ± 0.1	19 ± 2	150	2.9 ± 0.1	190 ± 20	15
Mut4	V193R	3.8 ± 0.4	9 ± 3	420	1.6 ± 0.1	140 ± 10	12
Mut4	V194H	3.5 ± 0.1	11 ± 2	320	1.4 ± 0.1	150 ± 10	10
Mut5	A199R	4.4 ± 0.1	9 ± 1	490			0.45c)
Mut6	G200K	3.4 ± 0.1	7 ±1	490	2.7 ± 0.2	120 ± 30	22
Mut7	192A/193R	2.9 ± 0.1	23 ± 2	130	4.8 ± 0.2	5 ± 1	960
Mut8	194H/199R	4.2 ± 0.1	12 ± 1	350	3.7 ± 0.1	71 ± 6	52
Mut9	194H/200K	3.1 ± 0.1	7 ± 1	440	2.6 ± 0.1	12 ± 1	217
**Mut10**	**193R/194H**	**4.4 ± 0.1**	**7 ± 2**	**630**	**4.6 ± 0.1**	**7 ± 1**	**660**
Mut11	192A/193R/194H	2.0 ± 0.1	16 ± 2	130	4.2 ± 0.2	3 ±1	1400
Mut12	193R/194H/199R	4.3 ± 0.1	7 ± 1	610	1.7 ± 0.1	4 ± 1	430
Mut13	193R/194H/200K	4.6 ± 0.2	4 ± 1	1200	3.3 ± 0.2	2 ± 0	1650
**Mut14**	**192A/193R/194H/199R**	**2.3 ± 0.1**	**11 ± 0**	**210**	**6.3 ± 0**	**3 ± 0**	**2100**
Mut15	192A/193R/194H/200K	3.1 ± 0.4	8 ± 1	390	4.0 ±.0.3	3 ± 1	1300

a) *v*_max_ and apparent *K*_m_ values were measured in cell free extracts at air-saturated oxygen levels

b) all indicated mutations confer to *Sm*NOX numbering including the starting methionine

c) as saturation with NADPH could not be achieved *v*_max_/app*K*_m_ was calculated from the initial linear section of the Michaelis-Menten curve

Ratios of NADPH and NADH activities and efficiencies measured from one cell free extract in parallel clearly indicated cofactor specificity changes between wild type and the variants. As expected, wild type *Sm*NOX showed only marginal activity with NADPH, while *Ls*NOX wild type showed NADPH oxidation, as reported [38]. In *Sm*NOX Mut1 and Mut2 (D192A and D192N) the negatively charged aspartate, which is known to be a key residue for NADH binding, is missing. In absence of the negative charge the activity with NADH was slightly reduced and higher NADH *K*_m_ values were detected. Remarkably, only by the exchange of the aspartate to non-polar or positively charged residues NADPH conversion rates increased to levels comparable to NADH rates, albeit with 10 fold higher *K*_m_ values.

The introduction of a positively charged residue next to the aspartate in Mut3 and Mut4 (V193R, V193H) without removal of the aspartate also enabled activity with NADPH although to a lower extent than the aspartate removal. NADH activity was not reduced in Mut3 and Mut4 compared to wild type *Sm*NOX containing extracts. The introduction of a positive charge was even more effective at position 200. NADH activity stayed unchanged compared to wild type for a Mut6 (G200K) variant, while NADPH activity already reached around 80% of the activity with NADH. A positively charged arginine at position 199 in Mut5 could not increase NADPH activity.

While single mutations already introduced quite high levels of NADPH activity, only the combination of mutations further decreased NADPH *K*_m_ values to values lower than 10 µM. The best combination for creating a mutant with high and matched NADH and NADPH activities at low *K*_m_ values is mutant Mut10 (V193R/V194H). Quadruple mutant Mut14 (192A/193R/194H/199R) showed the highest NADPH to NADH activity ratio of all mutants with a maximal NADPH activity three times as high as the NADH activity.

Sequence comparison of H_2_O_2_ and H_2_O forming NAD(P)H oxidase from *T. kodakarensis* (*Tk*NOX) and *Sm*NOX showed that *Tk*NOX features an arginine at positions equivalent to *Sm*NOX positions 194 and 199 and a lysine at the position equivalent to *Sm*NOX position 200 [45] (Figure 1). The results from the *Sm*NOX mutation study indicate that these positively charged residues might be responsible for making *Tk*NOX the only known bacterial wild type NOX showing higher activity with NADPH than with NADH.

Since Scrutton et al. [58] reported the first NAD(P)H specificity engineering of an enzyme, a vast number of enzymes have been mutated to alter cofactor specificities. The outcome of these mutation studies provided evidence that the effects obtained by site directed mutagenesis of positions known to be relevant for cofactor specificity, especially the aspartate or glutamate at the end of the second β-sheet, vary tremendously. Only in very few cases the catalytic efficiency of reactions with the originally disfavored cofactor could be increased to values of the same order of magnitude as for reactions with the originally used cofactor in the unmutated enzyme [10, 62–64]. For mutants in which the conserved aspartate or glutamate was exchanged to smaller non-polar residues, the efficiency with NADP^+^ ranged from 1/4000 to 1/3 of the efficiency of the unmutated enzymes with NAD^+^ [10, 65, 66]. Especially rare are examples of increased efficiency with one cofactor while keeping also the efficiency with the other cofactor high [10, 63]. In the outstanding case of *B. subtilis* lactate dehydrogenase the mutation of valine, the residue equivalent to *Sm*NOX V193, into arginine led to a 140 fold increased NADPH *k*_cat_ value. The increase in NADPH *k*_cat_ did not lead to a decrease but even to a four-fold increase in NADH *k*_cat_. *K*_m_ values stayed at wild type NADH level for both cofactors for the variant. In NADH oxidase from *L. plantarum* [37] the introduction of positively charged residues next to the conserved aspartate enabled *k*_cat_ values for NADPH of up to 69% of wild type level with NADH but decreased *k*_cat_ for NADH to 58% in variant G178R and to 16% in G178V/L179R. Interestingly, the single mutation G178R led to a low NADH *K*_m_ of 6 µM compared to 50 µM in the wild type form. NADPH *K*_m_ of the same variant was 490 µM and could be decreased to 9 µM in the G178V/L179R double mutant.

In summary, also in comparison to other enzymes, *Sm*NOX turned out to be an excellent choice for the generation of an NAD(P)H oxidase with comparable kinetic characteristics for both nicotinamide cofactors without drastic loss of activity or increase in *K*_m_ compared to the wild type enzyme.

### NADPH preferring SmNOX variants found in library screening

Around 3000 variants of a sequence saturation mutagenesis library (bought at SeSaM Biotech) built on *Sm*NOX 194H200K were screened for increase in NADPH/NADH activity ratios. We chose a starting point for the library without the well-studied mutation 193R as we rather aimed at finding new promising mutation combinations that were so far unknown to give high activities with NADPH.

**Figure 4:**
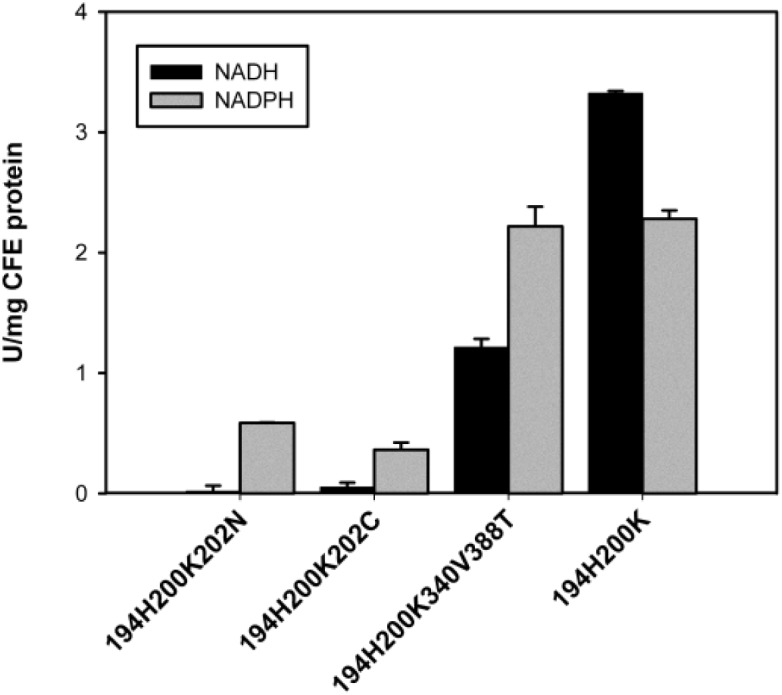
Cell free extract (CFE) activities of *S. mutans* NADH oxidase 2 variants found in a sequence saturation mutagenesis library by screening for improved NADPH/NADH activity ratios. The library was built on *Sm*NOX 194H200K and initial rates were measured employing 300 µM NADH or NADPH.

No variant with significantly increased NADPH activity without loss in NADH activity compared to *Sm*NOX 194H200K could be detected in the library screening. However, three mutants were identified with clearly increased NADPH/NADH activity ratio albeit with concomitant decrease in NADH activity. Figure 4 shows NADH and NADPH activity levels of crude lysates of mutant 194H200K202N, 194H200K202C and 194H200K340V388T compared to variant *Sm*NOX 194H200K. A 194H200K340V variant without 388T mutation was constructed to check for the influence of the mutation at position 340 without mutation at position 388. Variant 194H200K340V showed the same NADH/NADPH activity ratio as variant 194H200K340V388T. NADH activity was around 60% of NADPH activity in both cases. We conclude that mutation of position 340 has a high impact on cofactor specificity while mutation 388T probably has no influence on cofactor binding. Position 202 is located at the end of the loop which connects cofactor binding site strand βB and helix αB and starts after aspartate 192. The modeled *Sm*NOX structure indicates that residue 202 is positioned too far away from bound NAD(P)H in order to allow direct interaction with the cofactor (Figure 2). Strikingly, position 340 is located at the beginning of a β-sheet in close vicinity to the before described loop end. The *Sm*NOX wild type model indicates a potential hydrogen bond between Y202 and N340. In *Ls*NOX the equivalent positions are occupied by a tyrosine and a valine, which cannot form the hydrogen bond. We speculate that a presumably higher flexibility of the loop caused by removal of the hydrogen bond hampers the activity with the more structurally demanding NADPH less than activity with NADH.

### Purification of SmNOX and determination of kinetic values

**Figure 5:**
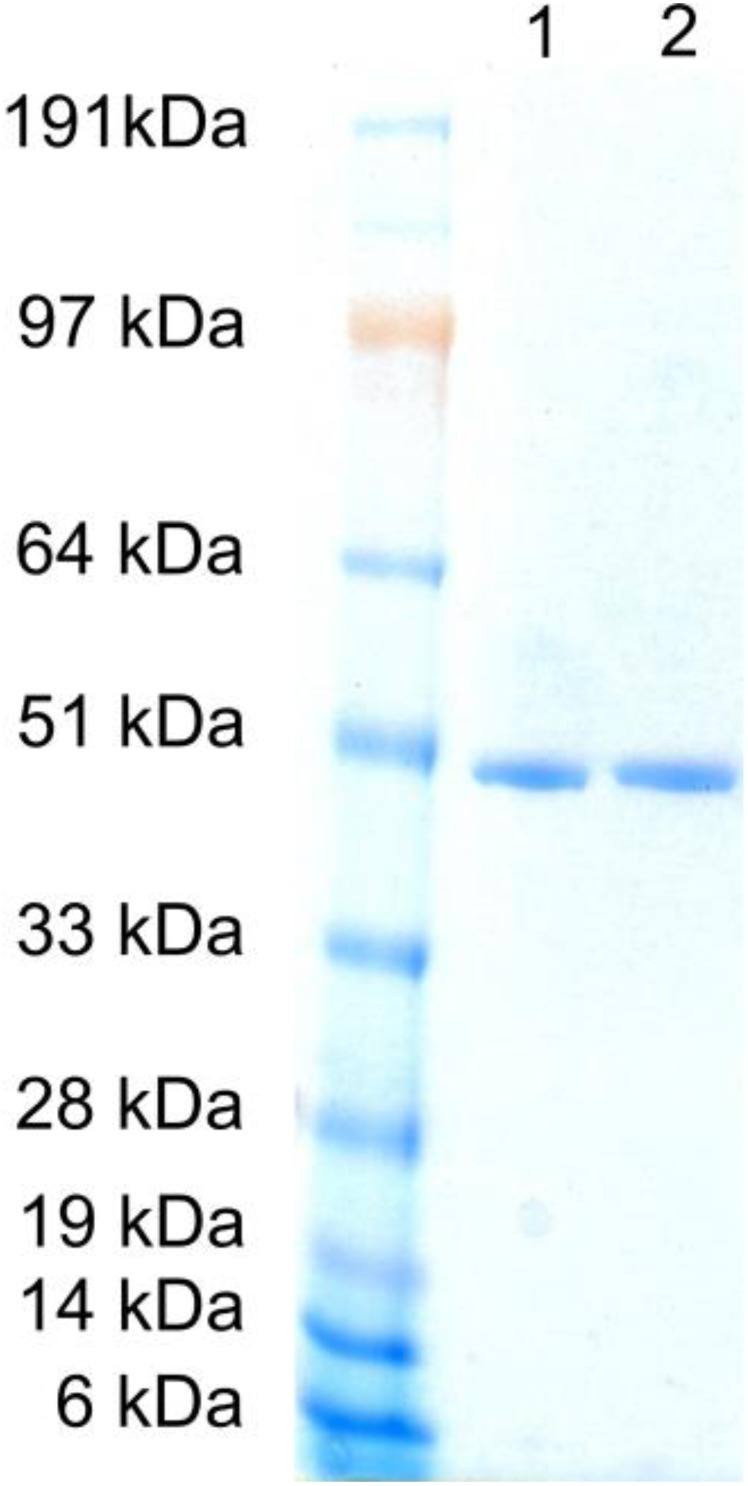
**SDS-PAGE of purified *S. mutans* NADH oxidase 2**. Standard: SeeBlue^®^ Plus2 Prestained Protein Standard; lane 1: wild-type; lane 2: 193R194H, SDS-PAGE was performed with an Invitrogen NuPAGE^®^ system with a 4-12% Bis-Tris Gel and Coomassie staining

*Sm*NOX wild type and variant 193R194H were recloned in vector pEHISTEV for expression with an N-terminal 6xHis tag. The enzymes were purified to apparent electrophoretic homogeneity by Ni-affinity chromatography as demonstrated in Figure 5. After purification the tag was cleaved off by treatment with TEV protease. Apparent kinetic values for NAD(P)H oxidation were determined in air saturated buffer as described in the methods section. *k*_cat_ and *K*_m_ values for *Sm*NOX wild type and mutant 193R194H are shown in Table 3. The *Sm*NOX wild type *k*_cat_ value corresponded to a specific activity of 44 U/mg. Higuchi et al. [51] reported a specific activity of water-forming *S. mutans* NOX 2 of 100 U/mg. The lower specific activity measured here was not unexpected due to a lower assay temperature than in the previous study. However, this lower activity could also be caused by enzyme deactivation during the tag cleavage procedure which included an overnight incubation at 4 °C. With *Sm*NOX mutant 193R194H now an efficient NADH oxidase is available which has very similar kinetic values for oxidation of NADH as well as NADPH.

**Table 3:** Apparent kinetic values and efficiencies for *Sm*NOX wild type and variant 193R194H from purified protein.

*Sm*NOX variant	NADH			NADPH		

	*k*_cat_ (s^-1^)	*K*_m_ (µM)	*k*_cat_/*K*_m_ (s^-1^µM^-1^)	*k*_cat_ (s^-1^)	*K*_m_ (µM)	*k*_cat_/*K*_m_ (s^-1^µM^-1^)
Wild type	36.8±0.3	7±2	5±2	n.d.	n.d.	n.d.
193R194H	25.5±0.9	9±2	3±1	20±2	6±2	3±1

n.d. not detected

### H_2_O_2_ production, influence of FAD and DTT

Possible H_2_O_2_ formation was analyzed by detecting H_2_O_2_ values after *Sm*NOX catalyzed total oxidation of up to 1 mM of NAD(P)H with *o*-dianisidine and HRP. For *Sm*NOX wild type with NADH 2.7% of the catalytic conversions led to H_2_O_2_ formation, for *Sm*NOX variant 193R194H with NADH in 3.3% of conversions and for NADPH in 4% of conversions H_2_O_2_ was formed. Other NOX enzymes were reported to lose FAD during purification [43]. Concentrated purified *Sm*NOX was clearly yellow, indicating that FAD was still bound. Initial rate measurements after 30 min pre-incubation in 25 µm or 250 µM and with addition of the same concentration of FAD to the assay showed an insignificant increase of activity for *Sm*NOX wild type and an insignificant decrease of activity for variant 193R194H. Several NADH oxidases have also been shown to be activated by addition of DTT [36, 47], probably by preventing the overoxidation of the catalytically active cysteine. *Sm*NOX activity was only slightly increased by addition of 5 mM DTT to the initial rate measurements.

### Conversion of 2-heptanol to 2-heptanone

**Scheme 1:**
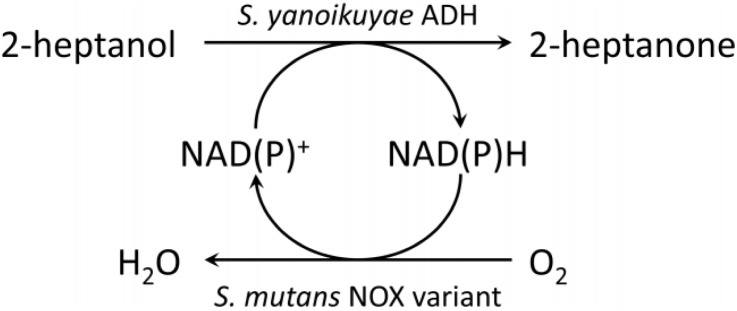
Cofactor regeneration by *S. mutans* NADH oxidase for 2-heptanol oxidation.

Application of water-forming NADH oxidases as cofactor recycling system coupled to a biocatalytic oxidation has so far been demonstrated for *L. sanfranciscensis* NOX [47, 67, 68], *L. brevis* NOX [69, 70] and *L. rhamnosus* NOX [48]. Here, we demonstrate the NADP^+^ regeneration by cofactor specificity engineered *Sm*NOX for 2-heptanol oxidation to 2-heptanone (Scheme 1). *Sm*NOX was applied in a coupled enzyme system with alcohol dehydrogenase from *Sphingobium yanoikuyae* (*Sy*ADH). 10 mM of 2-heptanol were converted to 2-heptanone in the presence of 100 µM of NADP^+^ with and without *Sm*NOX added to the assay. *Sy*ADH exhibits a low enantioselectivity for 2-heptanol [19] and therefore allows complete conversion if enough cofactor is supplied. NADP^+^ is cheaper than NADPH and therefore the preferred starting compound for cofactor recycling. Conversion rates are shown in Table 5. Nearly complete conversion could be achieved within 12 hours by addition of *Sm*NOX. Without *Sm*NOX, conversion rates around 1% as expected for a stoichiometric conversion of the 100 µM cofactor were found.

**Table 4:** Influence of FAD and DTT on *Sm*NOX, initial rates for NADH activity in 50 mM KP_i_ set to 100%.

*Sm*NOX variant	Buffer only	+25µM FAD	+250µM FAD	+5mM DTT
Wild type	100	108	112	104
193R194H	100	89	87	110

SD from at least triplicate measurements was always <16%

**Table 5:** Enzymatic 2-heptanol to 2-heptanone conversion with S. *yanoikuyae* ADH with and without NADP^+^ regeneration within 12 hours.

*Sm*NOX variant	2-heptanol	NADP^+^	U/mL *Sy*ADH	U/mL *Sm*NOX	% conversion	Cofactor turnovers
193R194H	10 mM	100 µM	1	2	97	97
-	10 mM	100 µM	1	0	1.2	1.6

## Conclusion

The NADH specific *S. mutans* NADH oxidase 2 belongs to the few NADH oxidases that produce water instead of hydrogen peroxide and is therefore well-suited to be used as cofactor recycling system. For general applicability *Sm*NOX was engineered towards efficient usage of both cofactors, NADH and NADPH. The dual cofactor specificity was achieved by introducing positively charged amino acid residues for increased NADPH binding, while still retaining the aspartate which facilitates NADH binding. *Sm*NOX variant V193R/V194H showed comparable high *k*_cat_ and *K*_m_ values for NADH and NADPH oxidation with only slight decrease in activity compared to *Sm*NOX wild type. NADPH specificity was also found to be increased by the mutation of Y202 or N340. These two residues form a hydrogen bond between the end of the nucleotide binding loop and a near-by positioned β-sheet in SmNOX wild type, which is destroyed by the mutations. *Sm*NOX variants were shown to be well active without a need to add FAD or DTT. Cofactor engineered *S. mutans* NAD(P)H oxidase 2 is therefore well suited for application as a versatile NAD(P)^+^ regeneration system, as was demonstrated for combination with *S. yanoikuyae* ADH for oxidation of 2-heptanol to 2-heptanone.
